# Recent Advances in Novel Lateral Flow Technologies for Detection of COVID-19

**DOI:** 10.3390/bios11090295

**Published:** 2021-08-25

**Authors:** Wesley Wei-Wen Hsiao, Trong-Nghia Le, Dinh Minh Pham, Hui-Hsin Ko, Huan-Cheng Chang, Cheng-Chung Lee, Neha Sharma, Cheng-Kang Lee, Wei-Hung Chiang

**Affiliations:** 1 Department of Chemical Engineering, National Taiwan University of Science and Technology, Taipei 106, Taiwan; letrongnghia720@gmail.com (T.-N.L.); hchang@gate.sinica.edu.tw (H.-C.C.); ns19jaipur@gmail.com (N.S.); cklee@mail.ntust.edu.tw (C.-K.L.); 2 GENTIS JSC, 249A, Thuy Khue, Tay Ho, Hanoi 100000, Vietnam; minhphd@gmail.com; 3 Institute of Biotechnology, Vietnam Academy of Science and Technology, 18 Hoang Quoc Viet, Cau Giay, Hanoi 100000, Vietnam; 4 Institute of Biological Chemistry, Academia Sinica, Taipei 115, Taiwan; fionako9220@gmail.com (H.-H.K.); chengung@gate.sinica.edu.tw (C.-C.L.); 5 Institute of Atomic and Molecular Sciences, Academia Sinica, Taipei 106, Taiwan

**Keywords:** COVID-19, SARS-CoV-2, lateral flow assay, point-of-care testing, artificial intelligence

## Abstract

The development of reliable and robust diagnostic tests is one of the most efficient methods to limit the spread of coronavirus disease 2019 (COVID-19), which is caused by the severe acute respiratory syndrome coronavirus-2 (SARS-CoV-2). However, most laboratory diagnostics for COVID-19, such as enzyme-linked immunosorbent assay (ELISA) and reverse transcriptase-polymerase chain reaction (RT-PCR), are expensive, time-consuming, and require highly trained professional operators. On the other hand, the lateral flow immunoassay (LFIA) is a simpler, cheaper device that can be operated by unskilled personnel easily. Unfortunately, the current technique has some limitations, mainly inaccuracy in detection. This review article aims to highlight recent advances in novel lateral flow technologies for detecting SARS-CoV-2 as well as innovative approaches to achieve highly sensitive and specific point-of-care testing. Lastly, we discuss future perspectives on how smartphones and Artificial Intelligence (AI) can be integrated to revolutionize disease detection as well as disease control and surveillance.

## 1. Introduction

The outbreak of coronavirus disease 2019 (COVID-19) caused by the severe acute respiratory syndrome coronavirus-2 (SARS-CoV-2) has had a detrimental effect on human health and interrupted regular social activities. Since the virus was first identified in Wuhan, China, in December 2019, the disease has spread globally and, according to the World Health Organization (WHO), has resulted in more than 4 million deaths (as of July 2021) [1]. COVID-19 is a potentially fatal respiratory illness with a broad spectrum of symptoms, which can include high fever, exhaustion, and a dry cough. These symptoms are the same as those caused by other respiratory illnesses (common cold, season allergies, influenza), making it difficult to distinguish from other ailments. Research has shown that patients who are suffering from other diseases, such as cancer, cardiovascular disease, and diabetes, or elderly patients are more likely to develop severe symptoms that require hospitalization [2].

The SARS-CoV-2 virus is transmitted through respiratory droplets, aerosols, or close contact with infected individuals. Recent studies demonstrate that infected patients, whether symptomatic or asymptomatic, may be contagious [3,4]. Mizumoto et al. reported that in the Diamond Princess cruise ship cluster, 18% of positive cases were recognized as asymptomatic [5]. In another cluster on an Argentinian cruise ship, 128 passengers tested positive for COVID-19. Among the COVID-19-positive patients, 104 positive cases (81%) were recognized as asymptomatic [6]. Therefore, accurate and effective diagnosis at COVID-19′s early stages is critical for reducing the risk of transmission, as it allows for quick isolation, contact tracing, and earlier treatment. An ideal diagnostic technique would be cost-effective, portable, rapid, and robust with high sensitivity and specificity [7,8]. This would allow for point-of-care (POC) testing and patient self-administration, resulting in rapid and adequate results and better epidemiological surveillance.

Currently available diagnostic techniques for COVID-19 are based on the detection of the viral gene, antigen, or human antibodies (serological test) and human metabolites [9,10,11,12,13,14,15,16]. Among these techniques, the detection of viral RNA sequences by reverse transcription polymerase chain reaction (RT-PCR), reverse transcription loop-mediated isothermal amplification (RT-LAMP), and reverse transcription quantitative polymerase chain reaction (RT-qPCR) have been the most reliable methods. RT-qPCR uses signal amplification to achieve a high degree of accuracy [17,18,19]. RT-LAMP is a newly established technique in which amplification occurs at a single temperature [20,21,22]. RT-qPCR is able to directly detect SARS-CoV-2 by monitoring the amplification of a targeted DNA molecule during the PCR [13]. Moreover, some novel technologies for detecting viral gene, such as next-generation sequencing (NGS) and Clustered Regularly Interspaced Short Palindromic Repeats (CRISPR), draw great attention due to their better accuracy and higher throughput [23,24]. However, these methods are expensive, time-consuming, and limited to well-trained professional operators. Therefore, they are often not amenable to extensive population-based or POC testing [25,26].

Virus antigens or host antibodies can also be detected serologically. The enzyme-linked immunosorbent assay (ELISA) is a rapid and inexpensive technique for detecting specific antibodies in blood samples. In a recent study, an ELISA test was used to detect human SARS-CoV-2 seroconverters [27]. This test enabled the detection of distinct antibody types as early as three days after the onset of symptoms. However, similar to RT-PCR techniques, the ELISA method also needs to be performed by well-trained personnel. It also relies on specialized equipment, making it difficult to use at POC testing.

Among available POC testing techniques, the lateral flow immunoassay (LFIA) has been extensively researched and used for COVID-19 diagnosis, owing to its low cost, speed, and accessibility [13,14,25]. To diagnose COVID-19, lateral flow tests combine SARS-CoV-2 pathogen assays with antibodies in patients. LFIA tests usually take around 10–30 min, while the conventional ELISA takes approximately 2–5 h. The sensitivity of COVID-19 detection by LFIA ranges from 61% to 88% (10 days after the first onset of symptoms) to 100% (after 3 weeks) [28,29]. However, early detection of the disease is a real challenge for LFIA, due to its low accuracy in detection. The accuracy of an LFIA device is evaluated in terms of its sensitivity and specificity. Thus, many efforts have been made to achieve higher sensitivity and specificity for SARS-CoV-2 detection in order to reduce false negative/positive predictive results. In a recent report, Xiang et al. showed that redesigned LFIA can obtain comparable sensitivity to ELISA [30]. Similarly, Smith et al. evaluated the sensitivity of the Quidel SARS Sofia rapid antigen flow immunoassay (USA) against RT-qPCR [31]. All tests achieved higher than 98% sensitivity to detect infected patients if tests were administered every three days. These evaluations confirmed the possibility of developing an ultrasensitive, highly specific LFIA for POC testing.

Since the use of RT-PCR, ELISA, and other techniques in SARS-CoV-2 detection has been discussed in a number of reviews elsewhere [9,11,13,14,15,17,18,25], in this article, we focus on recent advances in the development of novel lateral flow techniques as well as methods of increasing its sensitivity and specificity. Lastly, the next generation of POC testing for detecting COVID-19, such as smart phones and Artificial Intelligence (AI), will also be discussed.

## 2. Lateral Flow Technologies/Assays

Lateral flow technology (also known as lateral flow assay) plays a critical role in POC testing, as the technique is rapid, cost-effective, and can be operated by untrained personnel. In this article, depending on the analytes being targeted, lateral flow technologies can be classified as follows: lateral flow immunoassay (LFIA), nucleic acid lateral flow assay (NLFA), and nucleic acid lateral flow immunoassay (NALFIA). LFIA is able to detect antibodies/antigens, while NLFA uses a DNA or RNA probe to detect nucleic acid. Moreover, NALFIA uses both antibodies/antigens and nucleic acid as biomarkers for the detection of antigens/antibodies or amplicons [25].

## 3. Lateral Flow Immunoassay (LFIA)

Lateral flow immunoassay (LFIA), a qualitative chromatography, is a very simple, rapid, portable analytical platform that specifically targets the detection of antigens or antibodies [32,33]. LFIA was first reported in the early 1980s and commercialized in 1984 as the first product for a urine-based pregnancy test via the detection of human chorionic gonadotropin (hCG) [34]. Since then, over thousands of LFIA devices have been reported and applied toward the diagnosis and prognosis of various infectious diseases, cardiac diseases, tumors, pathogens, pesticides, toxins, and metal ions [35].

**Figure 1:**
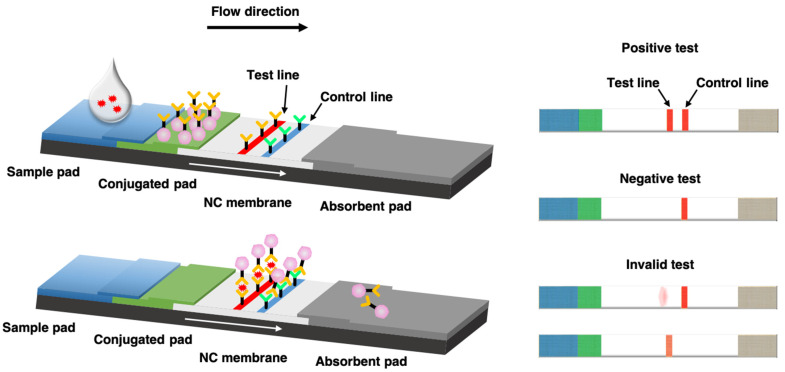
Schematic illustration of an antigen detection-based LFIA test.

LFIAs are typically composed of a sample pad, a conjugated pad, a nitrocellulose (NC) membrane, and an absorbent pad, as shown in Figure 1 [36,37]. As a paper-based chromatography, LFIA utilizes capillary forces to transport a fluid sample across various strip zones. Based on its design, LFIA could either detect the desirable antibodies or antigens through antibody–antigen interactions. The liquid sample is introduced to the sample pad of the strip and flows through the conjugated pad on which the targeted antibodies or antigens are conjugated with color or fluorescent reporter. Then, the complex reaches the NC membrane, where specific antibodies or antigens are fixed at a specified area, called the test line. Additionally, a control line is conjugated further along the NC membrane. If the targeted antibodies or antigens are contained in the sample, the forming reported antibody–antigen complex will induce color/fluorescent formation at the test line of the strip. Meanwhile, a proper formation of the control line indicates a properly conducted test. Additional test lines can also be immobilized to the NC membrane, allowing the detection of multiple antibodies/antigens in a single run. For quantification, the test strip is applied for optical/fluorescent reader for measurement of the color/fluorescent intensity [38,39].

**Figure 2:**
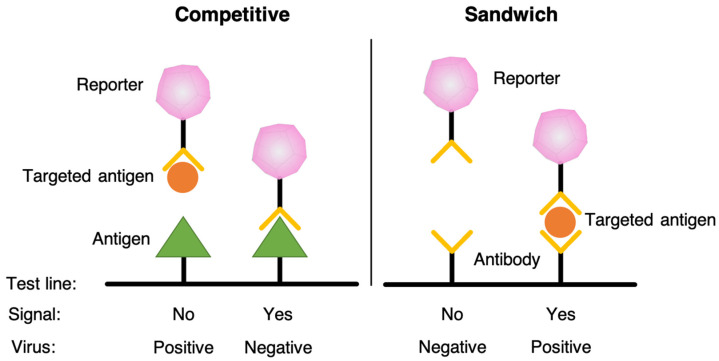
The competitive assay and sandwich assay models.

Currently, two common models of LFIA have been proposed, similar to ELISA, including competitive and sandwich models, as shown in Figure 2. The competitive assay that is designed for antigens has only one epitope that cannot simultaneously bind with two antibodies [40]. In the presence of analyte, the antibody–antigen interaction is formed, inhibiting the signal formation at the test line. Therefore, in the competitive test, the signal intensity is inversely proportional to the concentration of analyte in the sample. On the other hand, sandwich assay is used for analyte antigens, which has two epitopes that can simultaneously bind with two distinct types of antibodies, such as hCG used in pregnancy tests [41]. The targeted antigen is trapped between two antibodies at the test line, and therefore, the signal intensity is proportional to the amount of analyte in the sample.

Many liquid biological samples, including urine, saliva, perspiration, serum, plasma, whole blood, and other fluids, can also be analyzed with LFIA [42,43,44]. Numerous nanoparticles (NPs), including colloidal gold nanoparticles (AuNPs), quantum dots (QDs), fluorescent nanodiamond (FND), carbon black, selenium nanoparticles, and lanthanide-doped phosphors, have been employed as reporters. The conventional method uses AuNPs as a visual reporter for most assays [45,46]. AuNPs are easily functionalized. They also have enhanced stability, higher values for charge transfer, and good optical sensitivity. However, due to the technique’s low accuracy, significant improvements are required to make AuNPs suitable for diagnosing disease at its early stages.

## 4. Lateral Flow Technologies for COVID-19 Detection

### 4.1. Gene Detection

Using an NLFA, Yu et al. simultaneously detected three genes of the SARS-CoV-2 virus, including RdRp, ORF3a, and N protein gene [47]. The assay obtained a detection limit of 10 copies per test for each gene after 30 min. However, amplification using RT-PCR or some other technique was required prior to the NLFA process. In addition to high sensitivity and specificity, simultaneous detection was enabled to avoid false positive results due to the cross-reactivity of SARS-CoV-2, as well as false negative results due to the SARS-CoV-2 genome mutation. NFLIAs have also been studied for COVID-19 detection [48,49]. In another study, Wang et al. reported a nucleic acid immunoassay for detecting RNA of SARS-CoV-2 based on the binding of DNA probes to three genes (ORF3a, E protein gene, and N protein gene) without engaging in the pre-amplification process [49]. Then, SARS-CoV-2 antibodies were conjugated with europium chelate fluorescent nanoparticles and bound to the DNA–RNA hybrids. When testing with throat samples, the assay showed high sensitivity with a detection limit of 500 copies per mL in less than 1 h. Additionally, detecting three genes also helped avoid false positive results, making this technique a good candidate for POC testing. Several reports of SARS-CoV-2 are listed in Table 1.

### 4.2. Antigen Detection

Although many LFIAs for COVID-19 detection have been investigated and commercialized, there are only a few studies on antigen detection. The spike surface glycoproteins (S) and nucleocapsid proteins (N) of SARS-CoV-2 are the most commonly targeted antigens for antigen and serological tests. The characteristics of several reported LFIA devices for SARS-CoV-2’s antigen detection are shown in Table 1. For instance, Baker et al. used glycan as a binding agent to capture SARS-CoV-2 spike glycoprotein [50]. This LFIA device obtained 100% specificity with a detection limit of 5 μg mL^–1^. In another study, Diao et al. used N protein as a biomarker to detect SARS-CoV-2 in nasopharyngeal swabs and urine samples from patients with a suspected SARS-CoV-2 infection [51]. Carboxylate-modified polystyrene europium (III) chelate microparticles were used as fluorescent reporters. The test line and control line were constructed with the mouse anti-N protein of SARS-CoV-2 monoclonal antibody and the goat anti-rabbit IgG antibodies, respectively. The assay can be performed in 10 min with 100% specificity and 68% sensitivity compared to nucleic acid tests. In addition, latex beads are utilized as color reporters for N protein antigen detection with a detection limit of 0.65 ng mL^–1^ [52]. Overall, these assays are less sensitive than ELISA and RT-PCR tests. Hence, these tests are less popular than antibody detection-based LFIA and have a lower market share.

### 4.3. Antibody Detection

**Figure 3:**
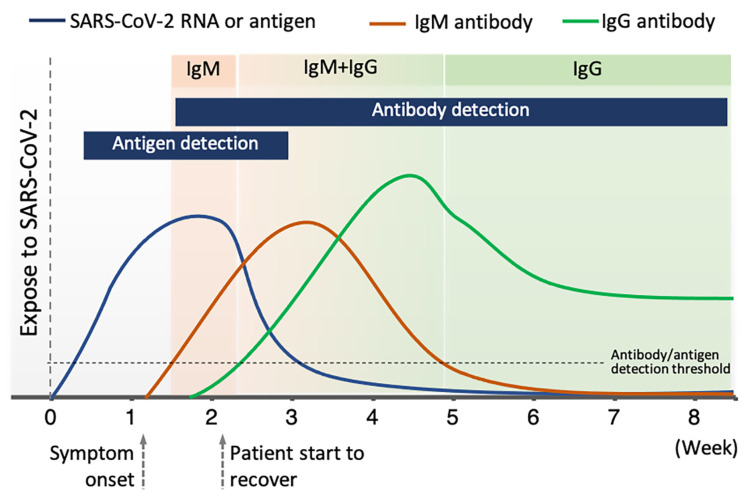
Levels of antibody and antigen at different clinical stage of COVID-19 disease.

Immunoglobulin M (IgM) antibodies and Immunoglobulin G (IgG) antibodies are two common types of antibodies generated by the human immune system. A number of LFIAs have been developed for detecting antibodies in the blood of patients who are exposed to the SARS-CoV-2 virus. However, focusing on antibody detection may lead to false negative tests when the disease is at its early stages. This is because in the days immediately following infection, antibodies might be below detectable levels, as shown in Figure 3 [68,69]. It has been demonstrated that 2–3 days after the onset of symptoms, the levels of IgM antibodies (as surveillance antibodies) rise, reaching its peak after 2 weeks [70]. Nevertheless, the levels of IgM will quickly decrease within 3 weeks. In contrast, the levels of IgG antibodies (as attack antibodies) increase 10–14 days after the first onset of symptoms. Then, the levels of IgG remain elevated for 4–5 weeks and decrease and stabilize after 5–6 weeks.

Thus, to avoid false negatives, the test needs to be performed at least 14 days after the first symptom. False positive results caused by cross-reactivity are also an important problem for these LFIA tests. For example, the similarity between the target SARS-CoV-2 antigen and other coronavirus antigens (such as SARS-CoV-1, MERS-CoV, HCoV-HKU1, HCoV-OC43, HCoV-NL63, and HCoV-229E) may impact the accuracy of LFIA tests [71]. The specificity of the antigen–antibody interaction is another crucial factor that directly correlates to the LFIA test’s efficiency. For instance, S1 subunits have higher specificity than N proteins for detecting SARS-CoV-2 antibodies [69]. Despite these limitations, many researchers and biotech companies have focused on antibody detection in COVID-19 diagnosis, which can be used to screen asymptomatic infected individuals to prevent possible spread of COVID-19. Several publications on the development of LFIAs that detect SARS-CoV-2 antibodies are also shown in Table 1.

**Figure 4:**
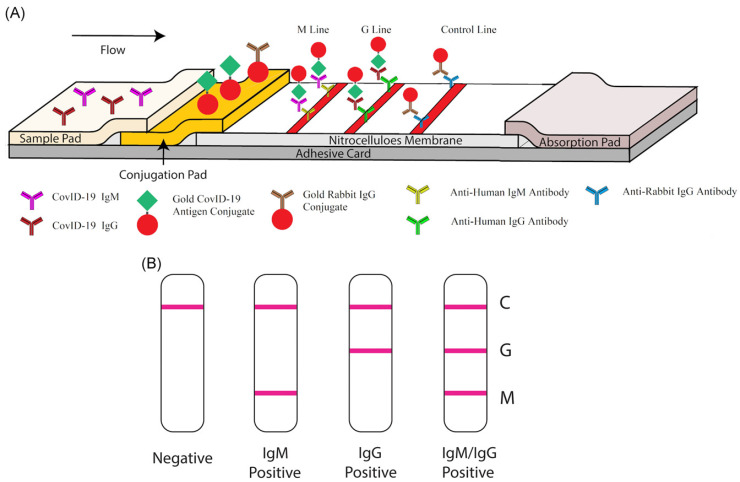
IgM–IgG combined antibody test for SARS-CoV-2 detection. (**A**) Schematic illustration of the LFIA device; (**B**) Results generated from the LFIA test. C: control line, G: IgG line, M: IgM line. Reprinted from [53].

In a recent study, Wen et al. put forward a method of rapid antibody detection for SARS-CoV-2. This process only takes 15–20 min and produces a visual readout [54]. In this study, AuNPs were used as reporters and were conjugated with mouse anti-human IgG (mAbs). This test had 69.1% sensitivity and 100% specificity. Furthermore, Li et al. combined the detection of IgG and IgM antibodies to facilitate higher sensitivity compared to a single antibody test [53]. As shown in Figure 4, a control line (anti-rabbit IgG), an IgG test line (anti-human IgG), and an IgM test line (anti-human IgM) were printed on the NC membrane. Once again, AuNPs were used as reporters. When run with a SARS-CoV-2 containing sample, IgG antibodies bound to the antigen-conjugated AuNPs and were captured at the IgG test line. Similarly, IgM-containing samples were captured at the IgM test line. In this work, 88.7% sensitivity and 90.6% specificity were obtained. The sensitivity of the IgG–IgM combined test showed higher sensitivity than single IgG or IgM detection.

**Figure 5:**
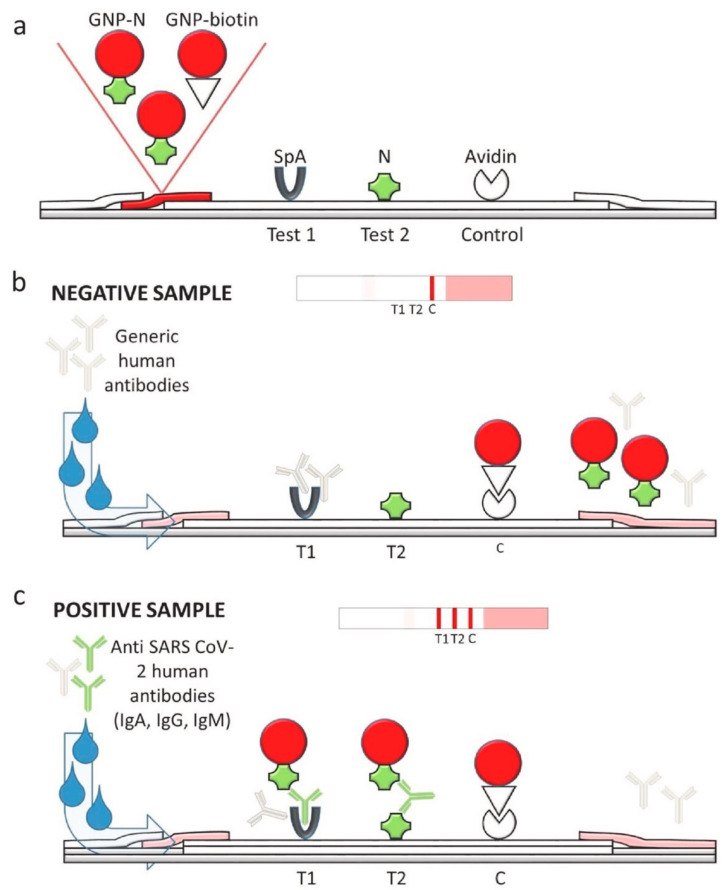
LFIA device for the rapid serological IgG, IgM, and IgA detection of SARS-CoV-2. (**a**) Protein A (SpA), SARS-CoV-2 N protein, and avidin were printed on the membrane for the T1 test line, T2 test line, and control line, respectively. N protein-labeled AuNPs and biotin-labeled AuNPs were used as reporters. (**b**) Negative test results consist of a single visible control line. (**c**) Positive test results showed three visible lines, indicating the simultaneous binding of antibodies (IgG, IgM, and IgA) to the T1 and T2 test line. Reprinted from with permission from [55]. Copyright 2020, Elsevier.

In order to achieve higher sensitivity, Calvalera et al. developed a multi-targeted LFIA that allows for the detection of total antibodies, including IgG, IgM, and IgA [55]. Staphylococcal protein A (SpA) and N protein of SARS-CoV-2 were used to construct the T1 and T2 test line, respectively (Figure 5). The control line consisted of avidin. AuNPs were labeled with N protein and biotin to act as reporters. SpA has been reported to bind with either human IgG antibody through Fc domain or IgM and IgA antibodies through Fab domains. Hence, the use of SpA and N protein antigen enables multi-target ability, and it results in a high sensitivity of 94.6% and 100% specificity. In addition, with the detection of IgA, the LFIA device seems to be a good early predictor of SARS-CoV-2, since IgA is known to be produced at detectable levels earlier than IgG and IgM [72].

Due to their rapid and low-cost properties, many LFIA devices have been available on the market, as shown in Table 2. Some examples of antigen-based detection devices are the CareStart COVID-19 Antigen test (Access Bio, Inc., Somerset, NJ, USA), Siofia 2 Flu + SARS antigen flow immunoassay (Quidel Corporation, San Diego, CA, USA), and BinaxNOW COVID-19 Ag Card (Abbott Diagnostics Scarborough, Inc., Scarborough, ME, USA). These test kits have been issued Emergency Use Authorization (EUA) by the US Food and Drug Administration (FDA), and they have a detection limit ranging from 4.17 × 10^5^ to 22.5 TCID_50_ mL^–1^ (TCID_50_: 50% tissue culture infective dose). The advantages of antigen detection kits are their low-cost, fast processing time (less than 20 min), high sensitivity (84–97.6%), and specificity (100%) [73]. In a recent study, Smith et al. demonstrated that the Quidel SARS Sofia antigen flow immunoassay (USA) showed similar sensitivity and specificity to RT-qPCR, which is very promising [31]. However, these test kits require a sample preparation step, the use of a specific instrument, or need to be performed by a trained operator, which limits their large-scale application.

In a comparison study, the average sensitivity of commercial kits (≈65%) was lower than the sensitivity of laboratory-based kits (≈88%) and of other serological methods (>80%) [87]. Moreover, although the claimed sensitivity and specificity of some commercial kits are high, the clinical accuracy of COVID-19 diagnosis is much lower, with the positive predictive value ranging from 11% to 50% [88]. In addition, LFIA detection devices are also associated with other challenges relating to difficulties controlling the fluid velocity and capillary force; the interferent porous membrane; the analysis time; and the sample nature [89,90]. Therefore, further efforts are needed to enhance the sensitivity and specificity of LFIA and ensure LFIA’s practical application in disease control and surveillance.

## 5. Enhancement of Sensitivity and Specificity

During the COVID-19 pandemic and beyond, the need for low-cost, simple, rapid, and highly accurate methods of disease detection is urgent. However, false negative and false positive test results due to low sensitivity and specificity make it difficult for lateral flow devices to detect disease at its early stages. Therefore, results of lateral flow devices should be confirmed with RT-PCR before they are used to inform decision-making surrounding isolation and treatment. Up to now, many efforts have been made to enhance the sensitivity and specificity of lateral flow technologies. Several methods have been developed, such as sample pre-concentration and amplification, signal enhancement using nanoparticles or an external signal reader, optimizing assay time, and the use of high affinity agents [88].

### 5.1. Sample Pre-Concentration and Pre-Amplification

If the same quantity of sample is used, conventional lateral flow technologies only achieve an average sensitivity of 66%, which is much lower than other serological assays. However, pre-concentration of the sample, before it is processed with lateral flow test, can significantly improve the assay’s sensitivity. Sharma et al. have used a magnetic field to pre-concentrate the analytes from the sample matrices to achieve 10 times higher sensitivity [91]. Furthermore, Mashayekhi et al. proposed to add Triton X-114 to concentrate the proteins by forming a two-phase micellar system to increase detection limits [92]. In the detection of transferrin, the detection limit of the lateral flow assay was significantly improved from 0.5 to 0.05 μg mL^−1^. By changing the volume ratio of these two micellar phases, these methods could potentially be leveraged to detect other proteins. In addition, the antigen–reporter complex can also be concentrated during the lateral flow assay running process by applying an electric field. The so-called isotachophoresis method allows for improved equilibrium binding and thus lowers the detection limit up to 400 times [93]. Pre-amplification is another excellent technique for increasing sensitivity. Amplifying DNA/RNA targeted samples by PCR prior to the lateral flow assay process can significantly boost lateral flow assay sensitivity up to RT-PCR’s sensitivity level [94]. However, the PCR amplification technique requires expensive instruments and well-trained personnel. Although sample enrichment methods can help increase sensitivity by ten to hundreds of times (even reaching ultrasensitivity), these methods still require additional equipment, extra preparation steps, or prolong the testing period, making it difficult for them to use for POC testing.

### 5.2. Signal Enhancement

Signal enhancement for lateral flow assays involves either the development of a new optical reporter system or utilizing an external signal reader to amplify the signal intensity and contrast. AuNPs with a nominal size around 20–40 nm have been widely used for conventional lateral flow assays. So far, most LFIAs for COVID-19 detection were developed using traditional AuNPs, but their sensitivities were not very high. In recent years, fluorescent nanoparticles have been increasingly applied in disease diagnosis and are promising alternative reporters, as their unique chemical and optical properties mean that they have the potential to enhance the sensitivity of lateral flow assays. Many fluorescence nanoparticles were utilized for SARS-CoV-2 detection, including QDs [95], FNDs [96], selenium nanoparticles [65,97], up-converting phosphor particles [98], lanthanide-doped nanoparticles [64], and aggregation-induced emission (AIE) nanoparticles [66].

#### 5.2.1. Gold Nanoparticles (AuNPs)

**Figure 6:**
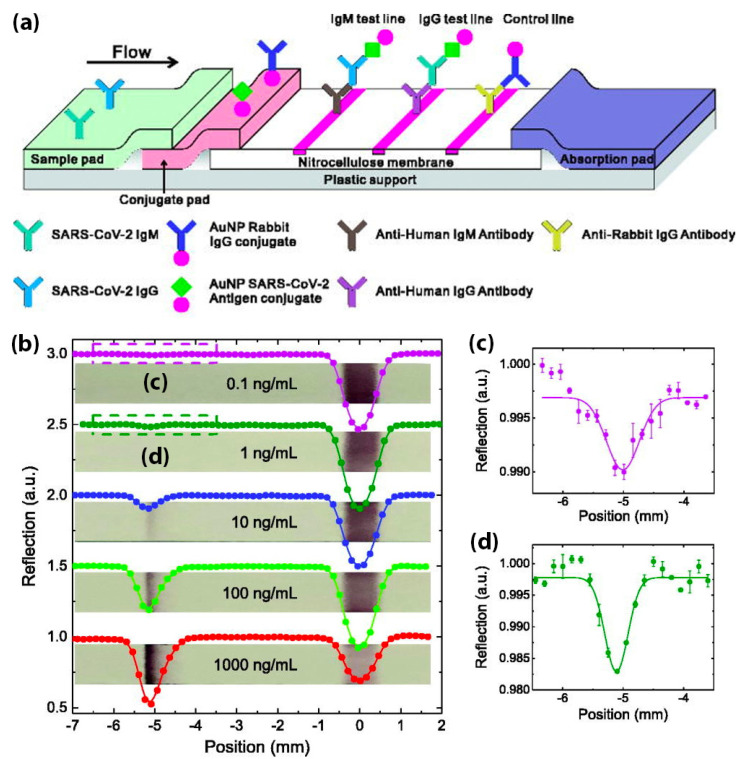
(**a**) AuNPs-based LFIA device for the detection of SARS-CoV-2 antibodies. (**b**) IgG concentrations on LFIA test strip from 1000 to 0.1 ng/mL, bottom to top. Dips at ≈−5 mm correspond to the IgG test lines and at ≈0 mm correspond to the control lines. (**c**,**d**) expansion spectra of 0.1 ng/mL and 1 ng/mL and Gaussian fittings (solid line). Reprinted from [58], with the permission of AIP Publishing.

Modifying the size or structure of conventionally used AuNPs can achieve higher sensitivity. The optical signal of gold nanoparticles in colorimetric lateral flow assay can be amplified through the deposition of silver, gold nanoparticles, and enzymes [90]. For example, Liu et al. used AuNPs-decorated silica nanorods as visual reporters for the detection of rabbit IgG [99]. The lateral flow assay strip had a detection limit that was 50 times lower than conventional AuNPs. In another study, Liu et al. coated AuNPs in polystyrene latex microspheres (PS) to increase the sensitivity for determining influenza virus H3 subtype [100]. Along with the sandwich format, the use of AuNPs-PS as a reporter for influenza virus detection can achieve 64 times higher sensitivity than that of 10 nm AuNPs. In addition to changing AuNPs’ size and structure, a photon-counting approach is another method of enhancing lateral flow assay’s sensitivity and detection limit. In a recent study, Peng et al. utilized a simple laser optical system to quantify SARS-CoV-2 IgG/IgM antibodies on a traditional AuNPs-based lateral flow assay with higher sensitivity, as shown in Figure 6 [58]. The LFIA strip was generally constructed with an IgG test line, an IgM test line, and a control line. Rabbit IgG-conjugated AuNPs and SARS-CoV-2 spike protein-coated AuNPs were used as reporters. For quantification, a 532 nm laser was directed at the LFIA strip and subsequently captured by a photon detector through a two-lens imaging system. This laser readout system can provide a rapid quantification on a conventional LFIA with a sensitivity of 0.1 ng IgG mL^−1^ and a detection limit of approximately 4 × 10^8^ IgG molecules.

#### 5.2.2. Quantum Dots (QDs)

Quantum Dots (QDs), also known as fluorescent semiconductor nanocrystals, can be a potential avenue for creating a highly sensitive lateral flow assay due to their high brightness, non-photobleaching, chemical and thermal stability, and ease of surface modification [101,102]. Their size of QD ranges from 1 to 10 nm. As a result, they are well-dispersed in water and can also combine with biomolecules. However, the small size of QD has limitations for the large-scale production of QD-based lateral flow assay [63].

**Figure 7:**
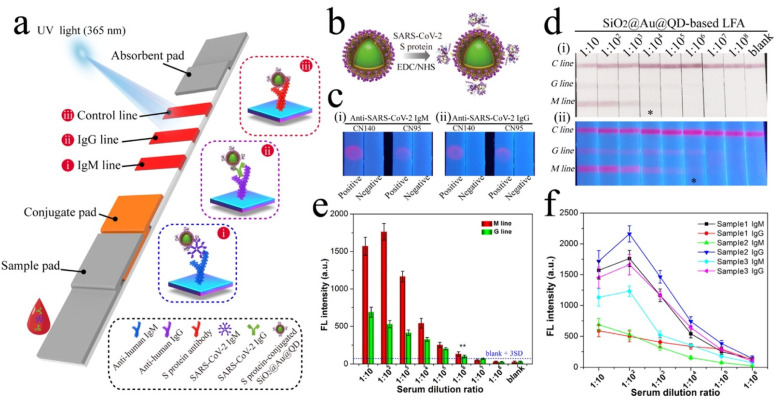
(**a**) A dual-mode SiO_2_@Au@QD-based LFIA biosensor for SARS-CoV-2 detection. (**b**) S protein-conjugated SiO_2_@Au@QDs were prepared by EDC/NHS coupling. (**c**) Optimization of LFIA NC membrane. (**d**) Photographs (**i**) and fluorescence images (**ii**) of the dual-mode LFIA for SARS-CoV-2-positive serum samples with different dilutions. (**e**) Corresponding fluorescence intensities of two test lines of the dual-mode LFIA. (**f**) Relationship of fluorescence intensity of test lines for three different positive serum samples with different dilutions. Preprinted with permission from [63]. Copyright © 2020, American Chemical Society.

Many QD-based LFIAs have been developed to achieve ultrasensitive detection [62,103,104,105]. In a recent study, Wang et al. reported a dual-mode LFIA for SARS-CoV-2 IgM/IgG detection based on QD nanobeads (Figure 7) [63]. Using the polyethyleneimine (PEI)-mediated electrostatic absorption method, QD nanobeads were composed of AuNPs (≈4 nm) and CdSe/ZnS-MPA QD layers outside the core of the SiO_2_ nanospheres (≈200 nm). Then, the SiO_2_@Au@QD nanobeads were conjugated with SARS-CoV-2 S protein to create a highly sensitive LFIA. The tests were carried out on 16 positive COVID-19 serum samples and 41 serum samples from other viral respiratory diseases. Through colorimetric and fluorescent signals, the QD nanobead-based LFIA achieved 100% sensitivity and 100% specificity for SARS-CoV-2 IgG detection. In another report, Liu et al. proposed a QD-based LFIA for SARS-CoV-2 detection method with 99% sensitivity and 100% specificity compared to the nucleic acid test [61]. Moreover, with QDs as a reporter, this LFIA utilized a portable fluorescent smartphone system, which is promising for ultrasensitive and highly specific POC testing.

#### 5.2.3. Fluorescent Nanodiamonds (FNDs)

Fluorescent nanodiamond (FND) is highly biocompatible, low in toxicity, and have physiochemical inert nanoparticles [106,107,108,109,110]. In addition, FNDs are an excellent alternative for LFIA reporters due to the high density of negatively charged nitrogen-vacancy (NV^–^) centers that act as built-in fluorophores [111]. The NV^–^ center is a six-electron system with two unpaired spins at ground state [112,113]. This color center is red fluorescent, photostable, and the spin states can be optically polarized through electronic excitation with green–orange light, resulting in a high-intensity fluorescence emission that reaches its maximum after excitation. However, under an external magnetic field, a decrease in fluorescence intensity is observed. This unique feature can be utilized as an effective method to detect FND by magnetic modulation of NV^–^ centers under the interference of high background conditions [111]. Moreover, the surfaces of FND can be chemically modified to contain emitters buried deep in a chemically inert matrix, so the optical properties are hardly affected by environmental changes (such as viscosity, pH, and ion concentration). Therefore, FND has been studied for biological applications such as nanoscale thermometry, drug delivery, and cell tracking [111,114,115,116,117,118,119], and it is a promising reporter for LFIA.

In a study, Feuerstein et al. used different sizes of FNDs (200, 400, and 800 nm) to develop a lateral flow technology for the detection of Ebola virus [120]. FND was conjugated with anti-Ebola virus glycoprotein monoclonal antibodies for a sandwich-type lateral flow test. The near-infrared emission can be measured by an in vivo imaging system or an optoelectronic device (OED). The 200 nm FNDs had the highest sensitivity among the test samples measured by the OED. Moreover, the OED provided quantitative data in less than one minute and was estimated to measure up to 1440 tests each day on a single OED setting. Furthermore, Miller et al. reported a spin-enhanced lateral flow system by using a microwave field [121]. The fluorescent intensity can be modulated to separate the autofluorescence background signal. The FND-based lateral flow assay can provide 10^5^ times higher sensitivity than conventional AuNPs. Moreover, after the signal pre-amplification process, the test can detect a single copy of HIV-1 RNA. The method also highlights the potential of FNDs as reporters for early disease diagnosis.

**Figure 8:**
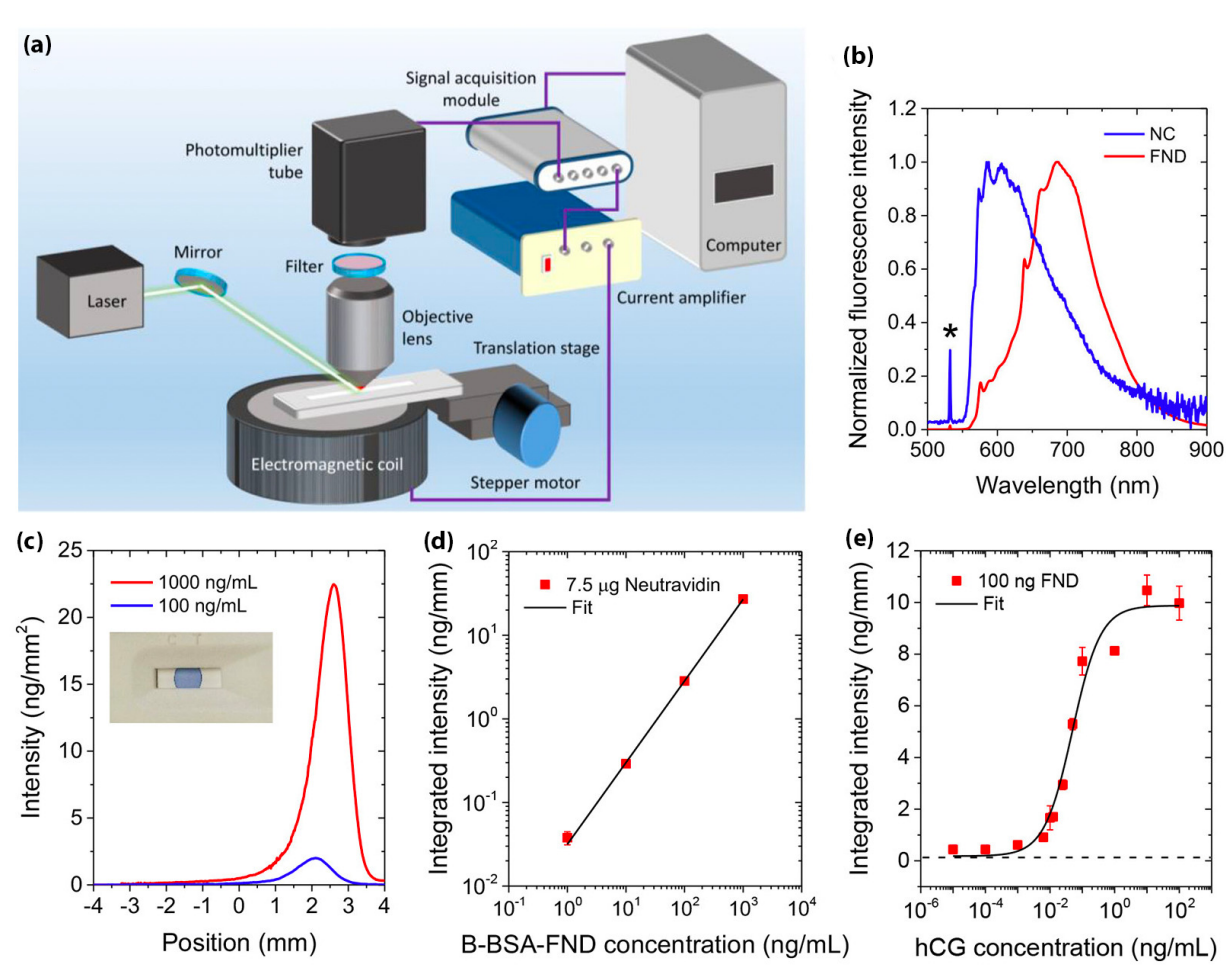
(**a**) Spin-enhanced lateral flow immunoassay (SELFIA) device. (**b**) Emission spectra of NC membrane and FNDs excited by a 532 nm laser. The asterisk (*) denotes unfiltered scattered laser light. (**c**) B-BSA-FNDs captured by the neutravidin bands formed on NC strips. The B-BSA-FND solution flowed from right to left. Inset: photo of a 1.5 μL trypan blue solution deposited on an NC strip to assist the assessment of the spot size of neutravidin. (**d**) Measured fluorescence intensities of B-BSA-FNDs captured by NC-bound neutravidin as a function of the B-BSA-FND concentration. (**e**) Sandwich SELFIA of hCG with anti-β hCG-coated FNDs and anti-β hCG deposited on NC strips. Reprinted with permission from [96]. Copyright © 2020, American Chemical Society.

More recently, Hui et al. utilized the unique magneto-optical properties of the NV^–^ centers in to develop a platform called Spin-Enhanced Lateral Flow Immunoassay (SELFIA) for ultrasensitive biomedical analysis (Figure 8) [96]. The SELFIA platform promises to effectively remove the conventional LFIA’s background fluorescence signals and obtain a lower detection limit compared to colloidal gold particles of similar size. In their work, the LFIA strip is generally made of NC membrane, which can interfere with fluorescent detection as a result of the photoexcitation process. Using FND as a reporter, SELFIA can offer background-free detection through magnetic modulation. The 100 nm FNDs were coated with biotinylated bovine serum albumin (B-BSA) and captured by neutravidin after the LFIA assays were processed. In addition, human chorionic gonadotropin (hCG)-β antibodies were coated with FND to detect hCG in a sandwich SELFIA. The FND on the NC membrane were detected at a particle density of 0.04 ng mm^−2^ (approximately 2 × 10^4^ particles per mm^2^). Moreover, by using larger size FND, the detection limit was reduced to 100 particles per mm^2^. The device offered ultrasensitive background-free detection using FND as a reporter, and it shows the promise of FND-based LFIA for detecting various viral diseases, including COVID-19.

Compared to AuNPs and QDs, there are only a few studies that have utilized FND as a reporter for LFIA devices so far. Further investigation of FND-based LFIA tests is necessary to determine its feasibility as an ultrasensitive and highly accurate method of disease detection. However, the development of new reporters has helped transform LFIA into a rapid and simple test without having to modify assay formats or steps.

### 5.3. Method for Improving Specificity

#### 5.3.1. Phage Display Technique for SARS-CoV-2 Antibody Selection

Specificity is another important factor that directly affects the accuracy of the lateral flow assay. Lateral flow assays for COVID-19 detection may be inaccurate due to the cross-reactivity of the SARS-CoV-2 virus with other coronaviruses. The cross-reactivity can reduce the specificity of the test, thus generating false positive results. To overcome this issue, phage display can be used to select SARS-CoV-2 antibodies with the strongest affinity. The phage display technique is a powerful method within the field of molecular biology that was awarded the 2018 Nobel Prize in Chemistry and has been widely used for the selection of antibodies, peptides, and disease-specific antigens [122]. In phage display, an exogenous DNA fragment encoding a protein of interest is inserted into a phage coat protein gene on the exposed surface, which is then capable of interacting with various external target molecules. This phenotype–genotype interaction enables researchers to isolate target-specific ligands [123].

**Figure 9:**
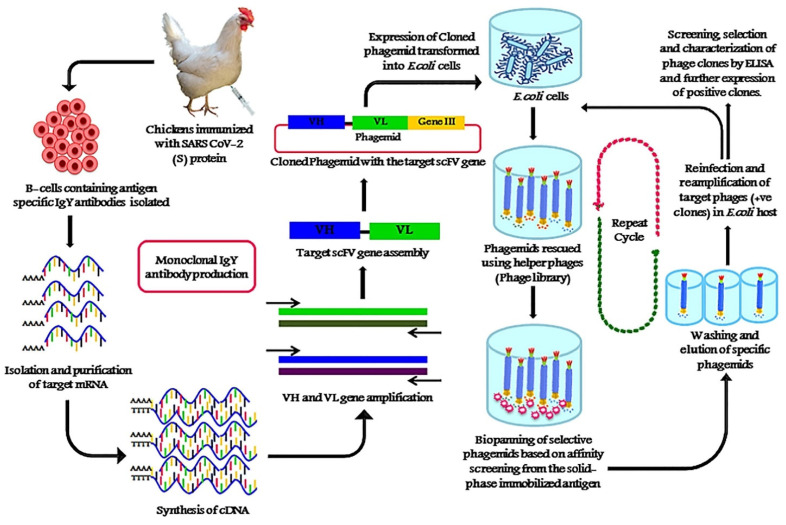
Production of anti-SARS-CoV-2 monoclonal scFv IgY antibodies against the spike protein (S) of SARS-CoV-2 antigen using phage display technology. Reprinted from [127]. Copyright 2020, with permission from Elsevier.

Phage display has been used to develop a variety of diagnostics and treatments, as well as in the investigation of antibody–antigen interactions, and epitope mapping for SARS-CoV-2 [122,124,125,126]. Using the phage display method, researchers are able to isolate SARS-CoV-2 monoclonal chicken egg yolk IgY antibodies (Figure 9) [127]. Compared to the commonly used IgG antibody for LFIA, the IgY antibody has a stronger binding affinity to the SARS-CoV-2 spike antigen. In addition, the production of IgY by phage display is cost-effective and profitable, since antibodies can be harvested largely from egg yolks. Moreover, IgY has been demonstrated to be heat resistant at a temperature range of 30–70 °C, stable at pH 3–11, and able to be stored for 6 months at room temperature or up to 10 years at 4 °C [128]. Therefore, due to its unique properties, IgY is a good candidate for LFIA and other diagnostics and therapeutics.

#### 5.3.2. CRISPR/Cas-Mediated Lateral Flow Nucleic Acid Assay

Clustered regularly interspaced short palindromic repeats (CRISPR) and CRISPR-related (Cas) have been used in many applications, including diagnostic, biosensing, imaging and led to the 2020 Nobel Prize in Chemistry. Therefore, many studies have applied CRISPR/Cas in COVID-19 detection [14,129,130]. In conjunction with lateral flow assay, Wang et al. developed a method called CRISPR/Cas-mediated lateral flow nucleic acid assay (CASLFA) using lateral flow assay incorporated with a Cas9 effector [131]. Within 1 h, the CASLFA can achieve a low detection limit (near to PCR level) with 100% specificity, indicating that CASLFA is a good candidate for further POC testing. However, more studies are required to optimize the CRISPR/Cas recognition step and assay time.

#### 5.3.3. Minimizing Non-Specific Binding

Specificity can also be enhanced by minimizing non-specific binding and non-specific interactions of the reporter to the targeted analytes and the membrane [88]. To reduce non-specific binding, a pre-filtration or centrifugation step can be applied to remove undesirable substances in the whole blood [132]. Optimizing reporter size and concentration and blocking the conjugated reporter by surface modification can also help minimize non-specific binding. Several proteins, sugars, and PEG polymer can be a surface coating or chemically conjugated to the reporter to enhance stability [90]. In addition, the running buffer also strongly affects the specificity of tests. Surfactants can help reduce non-specific binding; however, in high concentrations, it also reduces specific binding [88]. The pH and ionic strength of the buffer solution also need to be considered when optimizing the running buffer. A summary of methods for enhancing the sensitivity and specificity of lateral flow assays can be found in Table 3.

## 6. Role of Smartphones in Disease Control and Surveillance during the COVID-19 Pandemic

**Figure 10:**
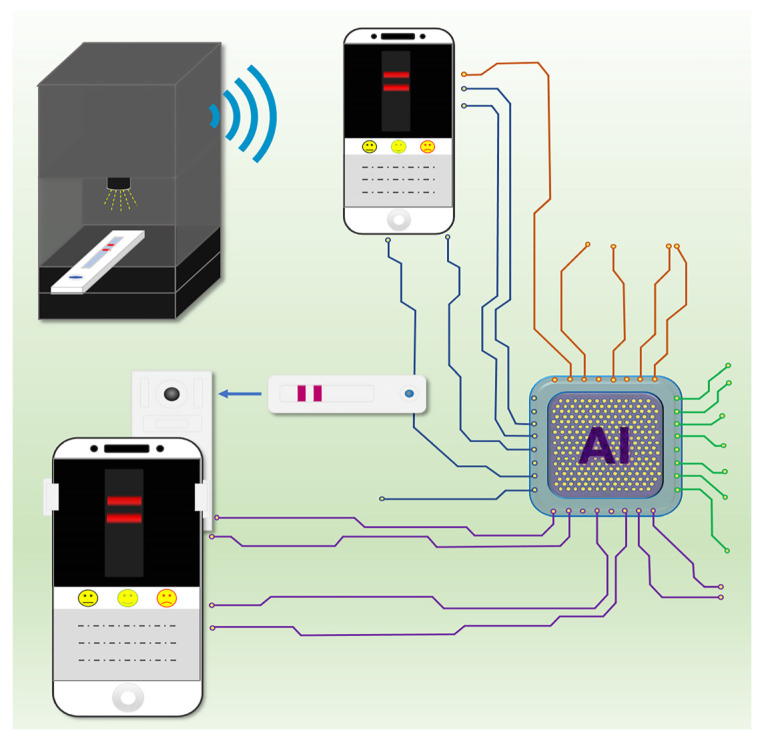
Schematic diagram of a portable AI-aided smartphone LFIA system for COVID-19 detection [61,133,134].

Recent smartphone generations are equipped with a powerful internal camera that can assist in POC testing when proper design software is implemented. Several applications that can assist in immunochromatography have been developed. These programs are capable of processing images to achieve quantitative analysis without any external equipment [33]. However, these methods may provide low reproducible results due to the interfering of light, camera position, or even the steadiness of the camera operator. For this issue, AI could be an excellent solution. Recently, Mendels et al. applied AI to improve test result interpretations of conventional LFIA assay for COVID-19 detection [133]. By developing their smartphone app, xRCovid, they largely removed human errors using machine learning. Finally, they claimed that this app achieved 99.3% precision compared to eye reading and is therefore beneficial for POC testing in the future. In addition, smartphones can be used for fluorescent reporter-based lateral flow assay, which usually provides higher sensitivity testing. By using a special lens or accessories, the device is portable, and can be used at the patients’ side for providing high accuracy and performance. Recently, Liu et al. reported a portable fluorescence smartphone system for ultrasensitive detection of IgM/IgG to SARS-CoV-2 [61]. Among 100 COVID-19 positive samples and 450 healthy blood samples, the QD-based LFIA for the detection of SARS-CoV-2 IgM and IgG antibodies can achieve 99% sensitivity with only 0.22% cross-reactive results. Moreover, AI has also been integrated with LFIA. The LooK SPOT AI COVID-19 Antigen Rapid Test system (Laipac Technology Inc., Canada) was approved by European CE-IVD as a LFIA diagnostic device that targets N protein antigen in nasal swabs [134]. Smartphone–AI-aided detection can quantitatively detect the signal intensity at a low level for reducing misdiagnosis. Test results can be provided in 5–8 min with a high sensitivity of 97.4% and a specificity of 98.3%. Figure 10 shows a schematic diagram of a portable AI-aided smartphone LFIA system for COVID-19 detection based upon the above-mentioned research work [61,133,134].

## 7. Conclusions

**Figure 11:**
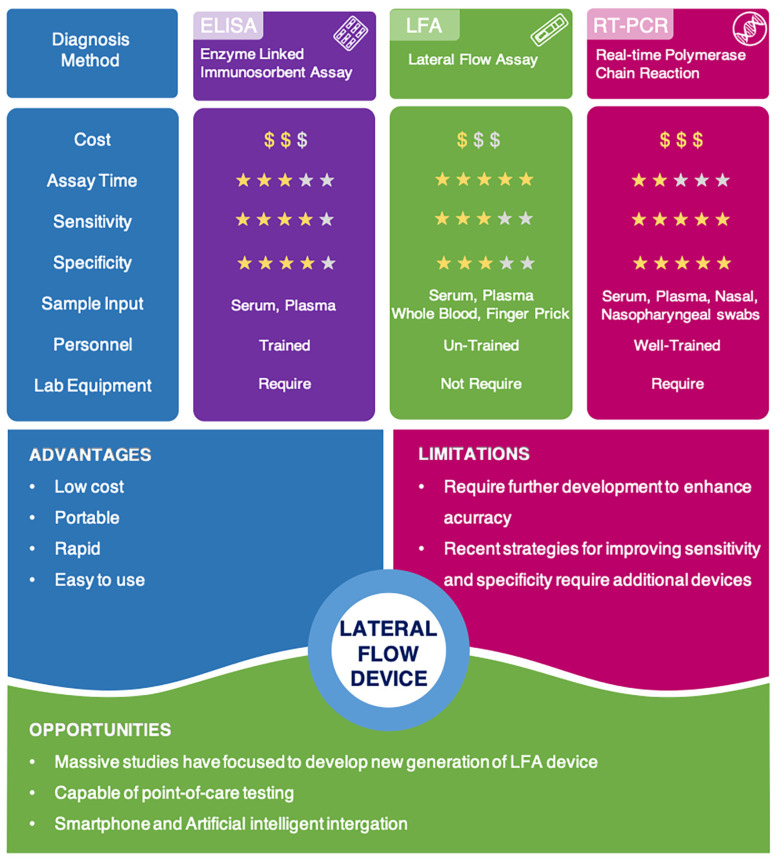
Comparison of current COVID-19 detection methods and advantages, limitations, and opportunities of lateral flow technologies.

Lateral flow technologies developed during the COVID-19 pandemic are portable, fast-acting, inexpensive, and easy to use, and therefore, they are becoming one of the most suitable techniques to practice POC testing. A comparison of recent COVID-19 detection methods has been described in Figure 11. Although false negative and false positive issues limit their clinical use, researchers around the world have worked together to improve the efficiency and accuracy of lateral flow tests in the hopes of creating a universal test for COVID-19. This article has provided an overview of lateral flow assay testing developments as well as detailed information about how their sensitivity and specificity can be improved. So far, many reports mentioned in this review were able to obtain comparable sensitivity and specificity testing at similar ELISA or even RT-PCR levels. However, most of these methods require the use of an external signal reader instrument. To overcome this issue, smartphone and AI integration can be mobilized in future POC testing.

## 8. Future Perspectives

Nowadays, smartphones are indispensable personal devices. As a result of their connectivity, Global Positioning System (GPS), and computational capabilities, smartphones can provide significant improvements to contact tracking and tracing, patient isolation, and monitoring for disease control and surveillance during a pandemic. Smartphones have been used as infectious diseases monitoring tools by identifying individuals who have been in contact with a patient. Finally, AI can also be used for analyzing every day test results, which is something that has been demonstrated to be a sufficient strategy for disease surveillance through accurately identifying infected individuals. In addition to lateral flow assays, many other diagnostic methods have been incorporated with smartphones and AI for POC testing, such as RT-PCR, CRISPR/Cas, chest computed tomography and paper microfluidic device [39,135,136,137,138,139,140]. These developments have revolutionized disease diagnosis by offering an approach to POC testing that is fast, accurate, cheap, and easy to use.

## Figures and Tables

**Table 1:** Selected laboratory prototypes of lateral flow tests for COVID-19 detection.

Type	Reporter	Biomarker	Detection Method	Detection Limit	Sensitivity	Specificity	Test Time	Ref.
Gene detection	Cy5	RdRp, ORF3a, N gene	Fluorescence	10 copies per test	100%	99%	30 min	[47]
	Crimson red-coated polymer NPs	ORF1ab, N gene	Visual/optical reader	12 copies per reaction	100%	100%	1 h	[48]
	Europium chelate NPs	E gene, N gene	Fluorescence	500 copies per mL	100%	99%	<1 h	[49]
Antigen detection	Glyco-AuNPs	S proteins	Visual/optical reader	5 μg mL^–1^	-	100%	5–30 min	[50]
	Carboxylate-modified polystyrene europium (III) chelate microparticles	N proteins	Fluorescence	-	68%	100%	10 min	[51]
	Latex bead	N proteins	Visual/optical reader	0.65 ng mL^–1^	-	-	-	[52]
Antibody detection	AuNPs	IgG, IgM	Visual	-	82.4%	100%	10 min	[30]
	AuNPs	IgG, IgM	Visual	-	88.7%	90.6%	15 min	[53]
	AuNPs	IgG	Visual	-	69.1%	100%	15–20 min	[54]
	AuNPs	IgG, IgM, IgA	Visual/optical reader	-	94.6%	100%	-	[55]
	AuNPs	IgA	Visual/optical reader, chemiluminescence	-	-	-	15 min	[56]
	AuNPs	IgM	Visual		100%	93.3%	15 min	[57]
	AuNPs	IgG, IgM	Visual, laser readout	4 × 10^8^ molecules	0.1 ng mL^−1^	-	-	[58]
	AuNPs	IgG, IgM	POC testing system	-	96.6%	100%	15 min	[59]
	AuNPs	IgG, IgM, IgA	Visual, lateral flow reader	-	100%	98.2%	30 min	[60]
	QDs	IgG, IgM	Portable fluorescence smartphone system	-	99%	100%	15 min	[61]
	QD nanobeads	Total antibodies	Fluorescence	-	97.1%	100%	15 min	[62]
	SiO_2_@Au@QD nanobeads	IgG, IgM	Visual, fluorescence	-	100%	100%	15 min	[63]
	Lanthanide-doped polystyrene NPs	IgG	Fluorescence	-	-	-	10 min	[64]
	Selenium NPs	IgG, IgM	Visual	-	94.7%	95.1%	-	[65]
	AIE NPs	IgG, IgM	Fluorescence	0.125 μg mL^–1^	95%	-	-	[66]
	SiO_2_@Ag nanocomposite	IgG, IgM	Surface-enhanced Raman spectroscopy	-	100%	100%	-	[67]

**Table 2:** Selected commercial lateral flow devices for COVID-19 detection.

Type	Test Kit	Sample Type	Biomarker	Detection Method	Sensitivity	Test Time	Characteristics
Antigen detection	BinaxNOW COVID-19 Ag Card, Abbott Diagnostics Scarborough, Inc. [74]	Nasal swab	N protein	Visual	22.5 TCID_50_/swab	15 min	POC testing; performance depends on following careful testing instructions
	CareStart COVID-19 Antigen test, Access Bio, Inc. [75]	Nasopharyngeal Swab	N protein	Visual	8 × 10^2^ TCID_50_/mL	10 min	Requires sample preparation step; operated by trained personnel
	Lumira Dx SARS-CoV-2 Ag Test, Lumira Dx UK Ltd. [76]	Nasal swab	N protein	Fluorescence	32 TCID_50_/mL	12 min	Requires Lumira Dx Platform; operated by trained personnel
	Sofia 2 Flu + SARS Antigen Flow Immunoassay, Quidel Corporation [77]	Nasal, Nasopharyngeal swabs	N protein	Fluorescence	4.17 × 10^5^ TCID_50_/mL	15 min	Detection of SARS-CoV-2, Influenza A Virus, and Influenza B Virus; limited to Sofia 2 Instrument; operated by trained personnel
Antibodies detection	Biohit SARS-CoV-2 IgM/IgG Antibody Test Kit, Biohit Healthcare (Hefei) Co., Ltd. [78]	Serum, plasma, venous whole blood (heparin, EDTA, and sodium citrate)	IgM and IgG	Visual	96.7%	10–20 min	Operated by trained personnel
	COVID-19 IgG/IgM Rapid Test Cassette, Healgen Scientific LLC [79]	Serum, plasma, whole blood	IgM and IgG	Visual	100%	10 min	Operated by trained personnel
	Diagnostic Kit for IgM/IgG Antibody to Coronavirus (SARS-CoV-2), Zhuhai Livzon Diagnostics Inc. [80]	Serum, plasma, venous whole blood	IgM and IgG	Visual	90.6%	15 min	-
	qSARS-CoV-2 IgG/IgM Rapid Test, Cellex Inc. [81]	Serum, plasma (EDTA or citrate), venous whole blood	IgM and IgG	Visual	-	15 min	Operated by trained personnel
	Sienna-Clarity COVIBLOCK COVID-19 IgG/IgM Rapid Test Cassette, Salofa Oy [82]	Serum, plasma, fingerstick whole blood	IgM and IgG	Visual	93.3%	15–20 min	Operated by trained personnel
	SARS-CoV-2 IgG IgM Antibody Rapid Test Kit, Lumigenex Co., Ltd. [83]	Serum, plasma, fingerstick whole blood	IgM and IgG	Visual	100%	15 min	Operated by trained personnel
	SARS-CoV-2 Antibody Test, Guangzhou Wondfo Biotech Co., Ltd. [84]	Serum, plasma, whole blood	IgM and IgG	Visual	86.4%	15 min	-
	RapCov Rapid COVID-19 Test, ADVAITE, Inc. [85]	Fingerstick whole blood	IgG	Visual	90%	15 min	Operated by trained personnel
	Rapid COVID-19 IgM/IgG Combo Test Kit, Megna Health, Inc. [86]	Serum, acid citrate dextrose plasma, fingerstick whole blood	IgM and IgG	Visual	100%	10–20 min	Operated by trained personnel

**Table 3:** Methods for enhancing sensitivity and specificity of lateral flow technologies.

	Strategy	Method
**Enhance sensitivity**	Sample enrichment	Pre-concentration: Filtration Centrifugation Magnetic pre-concentration Applying electric field PCR pre-amplification of nucleic acid analytes
	Signal enhancement	Nanomaterials as reporter: Modified AuNPs, quantum dots, fluorescent nanodiamond, selenium NPs, up-converting phosphor particles, lanthanide-doped NPs, aggregation-induced emission (AIE) NPs External reader: Optical reader Fluorescence Chemiluminescence Raman
**Enhance specificity**	Maximizing specific binding	High-affinity molecules Phage display technology for antibody selection CRISPR/Cas-mediated lateral flow assays
	Minimizing non-specific binding	Pre-filtration or centrifugation Optimizing size and concentration Surface modification with proteins, sugars, PEG. Optimizing running buffer Membrane blocking
